# Left atrial remodeling by MRI: comparison in patients with and without cardiovascular disease

**DOI:** 10.1186/1532-429X-16-S1-P152

**Published:** 2014-01-16

**Authors:** Dana C Peters, Bethlehem Mekonnen, Hamid Mojibian, Mark A Mareib, Gourg Atteya, Daniel Cornfeld

**Affiliations:** 1 Diagnostic Radiology, Yale School of Medicine, New Haven, Connecticut, USA; 2 Cardiology, Yale School of Medicine, New Haven, Connecticut, USA

## Background

Pre-existent scar in the left atrium (LA) is hypothesized to be the arrhythmic substrate of atrial fibrillation (AF)[1,2], and has been characterized in cardiac MR studies [3] using high resolution late gadolinium enhancement (LGE) [4]. Recent pathology studies show that LA fibrosis extent is similar in patients with AF vs. other cardiovascular disease (CVD) [5], e.g. valvular disease, ischemic and non-ischemic cardiomyopathies. Our aim was replicate these pathology findings by comparing patterns of enhancement on LGE in patients with and without CVD.

## Methods

Forty-eight patients were imaged at our center on a 1.5T scanner (MAGNETOM Espree, Siemens Healthcare, Erlangen), having good-quality LGE studies with visibly enhanced mitral valves, and no prior pulmonary vein isolation. Left atrial LGE was obtained using an ECG-triggered, navigator-gated 3D GRE inversion recovery (IR) sequence obtained 10-20 minutes after the administration of 0.2 mmol/kg of Gadobutrol, with spatial resolution of 1.5 × 1.4 × 3 mm3. A blinded assessment of enhancement/scar (0 = absent, 1 = present) was performed in an 18 segment model. Segments included 4 regions around each PV, and the LA posterior wall and inter-atrial septum. The total scar score was the % of enhanced segments. A chart review was performed to obtain clinical data.

## Results

**Figure 1:**
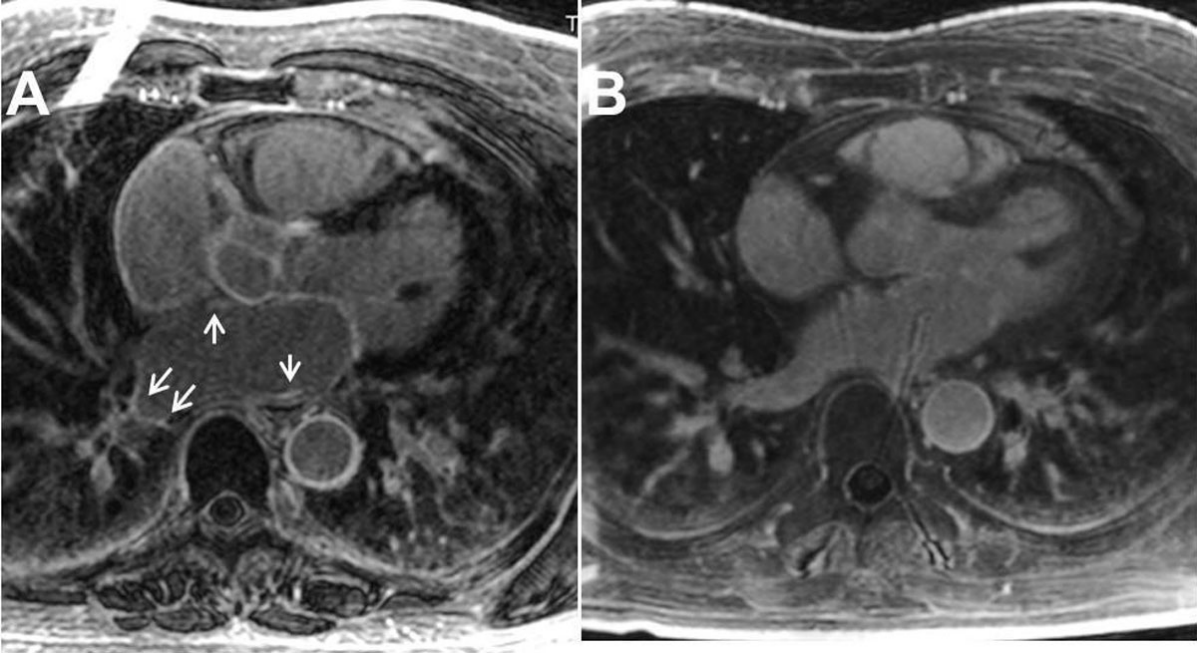
**A) 62 y.o. man with ventricual tachycardia, an enlarged right atrium and a scar score = 83% (see arrows)**. B) 58 y.o. man without CVD, and a scar score = 5%.

Figure 1 compares LGE images from our cohort. Patients with no CVD (N = 11, typically subjects with a family history of inheritable CVD) had a lower scar score than patients with CVD (N = 37, with a wide range of cardiovascular disease types) (19 ± 14% vs. 38 ± 21, p = 0.018). Six AF patients had a similar scar score to patients with other CVD (34 ± 21% vs. 39 ± 24%). Table 1 shows correlations between high scar score and right atrial enlargement, but no other analyzed variables. LA enlargement itself was associated with increased age (40 ± 19 vs. 55 ± 17, p = .046) and an insignificantly higher BMI (26.7 ± 4.0 vs. 29.7 ± 5.5, p = 0.14), similar to what has been reported previously [6].

**Table 1:** Characteristics for patients with high and low scar scores.

	All	High Scar Score	Low Scar Score
Age (years)	46 ± 19	46 ± 22	46 ± 16

Male	58%	54%	62%

LV EF	51 ± 14	49 ± 18	52 ± 8

LV EDV	158 ± 39	153 ± 48	163 ± 30

BMI (kg/m^2)	27 ± 6	27 ± 6	29 ± 6

RA enlargement (%)*	26%	44%	11%

LA enlargement (%)**	25%	35%	16%

Hypertension (%)***	43%	27%	60%

***p = 0.18, **p = 0.25, *p = 0.05, Fisher's exact test.

## Conclusions

This retrospective analysis found that LA LGE was greater in subjects with CVD vs. those with no CVD, corroborating pathology studies of LA fibrosis. LA LGE enhancement was associated with atrial enlargement. Non-invasive LGE provides a potentially powerful tool for determining clinical factors implicated in LA fibrosis, which would add to the understanding of its causes.

## Funding

NIH (NHLBI R21 HL 098573 & R21 HL103463).
